# Detection of RNA structures in porcine EST data and related mammals

**DOI:** 10.1186/1471-2164-8-316

**Published:** 2007-09-10

**Authors:** Stefan E Seemann, Michael J Gilchrist, Ivo L Hofacker, Peter F Stadler, Jan Gorodkin

**Affiliations:** 1 Division of Genetics and Bioinformatics, IBHV, University of Copenhagen, Grønnegårdsvej 3, DK-1870 Frederiksberg, Denmark; 2 Bioinformatics Group, Department of Computer Science, University of Leipzig, Germany; 3 The Wellcome Trust/Cancer Research UK Gurdon Institute, Cambridge, CB2 1QN, UK; 4 Institute for Theoretical Chemistry and Structural Biology, University of Vienna, Austria

## Abstract

**Background:**

Non-coding RNAs (ncRNAs) are involved in a wide spectrum of regulatory functions. Within recent years, there have been increasing reports of observed polyadenylated ncRNAs and mRNA like ncRNAs in eukaryotes. To investigate this further, we examined the large data set in the Sino-Danish PigEST resource  which also contains expression information distributed on 97 non-normalized cDNA libraries.

**Results:**

We constructed a pipeline, EST2ncRNA, to search for known and novel ncRNAs. The pipeline utilises sequence similarity to ncRNA databases (blast), structure similarity to Rfam (RaveNnA) as well as multiple alignments to predict conserved novel putative RNA structures (RNAz). EST2ncRNA was fed with 48,000 contigs and 73,000 singletons available from the PigEST resource. Using the pipeline we identified known RNA structures in 137 contigs and single reads (conreads), and predicted high confidence RNA structures in non-protein coding regions of additional 1,262 conreads. Of these, structures in 270 conreads overlap with existing predictions in human. To sum up, the PigEST resource comprises trans-acting elements (ncRNAs) in 715 contigs and 340 singletons as well as cis-acting elements (inside UTRs) in 311 contigs and 51 singletons, of which 18 conreads contain both predictions of trans- and cis-acting elements. The predicted RNAz candidates were compared with the PigEST expression information and we identify 114 contigs with an RNAz prediction and expression in at least ten of the non-normalised cDNA libraries. We conclude that the contigs with RNAz and known predictions are in general expressed at a much lower level than protein coding transcripts. In addition, we also observe that our ncRNA candidates constitute about one to two percent of the genes expressed in the cDNA libraries. Intriguingly, the cDNA libraries from developmental (brain) tissues contain the highest amount of ncRNA candidates, about two percent. These observations are related to existing knowledge and hypotheses about the role of ncRNAs in higher organisms. Furthermore, about 80% porcine coding transcripts (of 18,600 identified) as well as less than one-third ORF-free transcripts are conserved at least in the closely related bovine genome. Approximately one percent of the coding and 10% of the remaining matches are unique between the PigEST data and cow genome. Based on the pig-cow alignments, we searched for similarities to 16 other organisms by UCSC available alignments, which resulted in a 87% coverage by the human genome for instance.

**Conclusion:**

Besides recovering several of the already annotated functional RNA structures, we predicted a large number of high confidence conserved secondary structures in polyadenylated porcine transcripts. Our observations of relatively low expression levels of predicted ncRNA candidates together with the observations of higher relative amount in cDNA libraries from developmental stages are in agreement with the current paradigm of ncRNA roles in higher organisms and supports the idea of polyadenylated ncRNAs.

## Background

Genomic studies show that only a small proportion of transcribed RNAs represent messenger RNAs [1]. Less than 2% of the human genome codes for proteins, although a large fraction of the eukaryotic genome is transcribed [2-5]. In fact, the ENCODE Pilot Project provides evidence that almost the entire non-repetitive part of the human genome is transcribed in at least one of the two reading directions [6]. In agreement with this, in recent years, there has been reported an increasing number of functional non-coding RNAs (ncRNAs) [7]. The discrimination between functional ncRNAs and genomic transcription background is a complex problem [8] since ncRNAs do not present common primary sequence features. Furthermore, recent results show that very short ORFs may be translated to yield functional proteins [9], emphasizing that the absence of a long open reading frame alone does not necessarily imply that a transcript functions as ncRNA.

Most of the "house keeping" ncRNA families (tRNAs, rRNAs, snRNAs, snoRNAs) and a large class of regulatory RNAs (in particular miRNAs) have characteristic structures which perform an evolutionary conserved function. This property can be utilized in comparative genomics approaches to recognize such functional RNAs [10-13]. Such computational surveys have resulted in the prediction of many thousands of genomic loci with evidence for stabilizing selection of RNA structures [14-20].

Despite the relatively high false positive rates of ncRNA predictions by programs such as EvoFold, RNAz, and foldalign all these approaches yield clear statistical evidence that evolutionary conserved RNA structure is a pervasive feature of eukaryotic genomes. The experimental verification of the predictions is a complex issue. Northern blots, the method of choice for this task, require thousands of copies of the RNA molecule for a detectable signal. Non-coding transcripts, however, appear to be expressed at much lower levels than most protein-coding mRNAs, see e.g. [6]. Furthermore, some ncRNAs might only be expressed for a short time at a particular developmental stage or only in a very specific tissue. An extreme example is the microRNA *lsy*-6 in C. *elegans*, which is expressed only in a single neuron of the worm [21].

Expressed sequence tags (ESTs) represent short subsequences of transcribed RNAs. They are produced by an automated and cost effective sequencing mechanism that, however, results in low quality sequences which typically do not cover the complete transcript. The majority of EST data comprise poly(A)(+) RNAs since the cDNAs are obtained using a poly-T primer. Polyadenylation is often thought of as a characteristic feature of messenger RNAs (mRNAs). Mammalian transcriptomes, however, contain thousands of mRNA-like RNAs that are spliced but do not have appreciable ORFs or other evidence for protein coding capacity. This class of transcripts constitutes a significant fraction of the poly(A)(+) RNAs, see e.g. [1,22,23] and the references therein. Computational studies [12,16,17] showed that many of these "mRNA-like" ncRNAs, including prominent examples such as *H19 *and *Xist*, contain local, conserved secondary features, which hint at a functional role for the RNA itself.

Intriguingly, there is growing evidence of polyadenylation also for classical RNAs. The Gene Ontology has consequently been recently extended by the term GO:0043629 "ncRNA polyadenylation". Several examples come from yeast [24,25], polyadenylated snRNAs have been observed in *Dictyostelium discoideum *[26,27].

Recent studies identified a second nuclear poly(A) polymerase in yeast that is conserved through eukaryotes and tags aberrant non-coding RNAs for degradation [28]. In addition, a growing number of microarray surveys of different organisms, in which oligo(dT) oligonucleotides have been used to amplify the cDNA probes, have also yielded hybridization signals from ncRNA targets. Examples of this are whole-genome (tiling) microarray experiments using RNA from human [29], mouse [22] and arabidopsis [30]. Even though this method does not provide any direct evidence for polyadenylation, it indicates that the phenomenon of polyadenylated ncRNAs is more widespread than previously anticipated [31]. Therefore, it is of interest to search for both ncRNAs that are normally not polyadenylated and mRNA-like ncRNAs in poly(A)(+) EST libraries.

Here, we report of the computational detection of novel ncRNAs and structural RNA cis-regulating (UTR) elements in the EST libaries of the Sino-Danish pig genome project [32]. An automated pipeline EST2ncRNA was designed and applied to the assembled porcine EST data, which consists of 48,629 clusters (contigs) and 73,171 single reads (singletons). Contigs and single reads are collectively referred to as *conreads *[32] in the following. Predicted RNA structure candidates are further merged with expression information available in the PigEST resource [33] which contains an assembly of more than one million EST sequences. One-third of these originates from public available cDNA libraries and two-thirds originate from one normalized and 97 non-normalized cDNA libraries (35 tissues), of which 24 stem from developmental stages.

## Results

**Figure 1:**
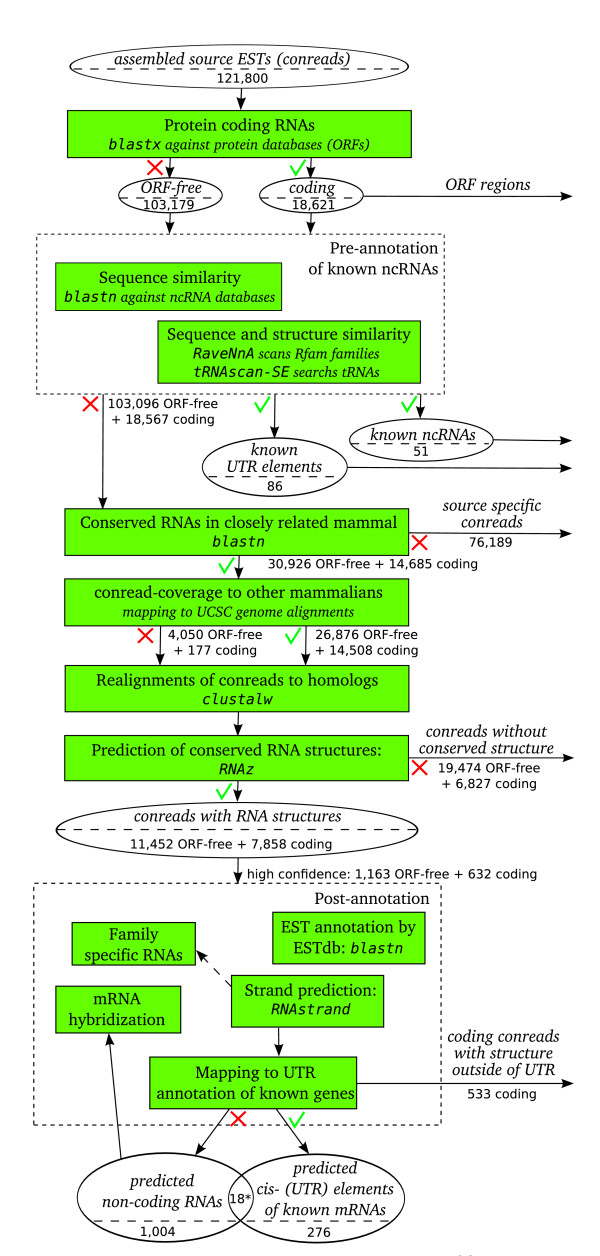
The flowchart illustrates the basic functionality of the EST2ncRNA pipeline. Furthermore, the number of PigEST conread candidates are shown for each step. The pipeline detects known and novel candidates of functional non-coding RNAs as well as cis-regulatory elements from large assembled EST datasets. In summary, the pipeline consists of four main steps: (1) pre-annotation of known ncRNAs, (2) homology search in other organisms, (3) prediction of thermodynamic stable and conserved RNA structures, and (4) post-annotation. A method to detect family specific RNAs in the post-annotation is RNAmicro for instance. The candidates for mRNA hybridization predicted by RNAduplex are not included in the publication. The sign × indicate sequences not matching a given step in the pipeline. The sign √ indicate the sequences which were matched. (*) 18 ORF-free conreads contain two independent RNA structures of which only one is similar to an human UTR. Therefore, these conreads are counted twice.

The EST2ncRNA pipeline (see Figure 1) was used to analyse the PigEST data. In the following we present our results in terms of the number of conreads that contain at least one high confidence RNA structure (RNAz classificator *p *> 0.9 and thermodynamical stability score *z *< -3), because only a small fraction of conreads contains two or more predicted structured loci. See Table 1 for an overall summary of detected known and novel non-coding RNAs and cis-acting RNA elements in the PigEST data. Furthermore, Figure 1 denotes the number of candidates at each step of the pipeline.

**Table 1:** Known and novel ncRNAs and UTR elements in PigEST data

	Method	Contigs	Singletons
ncRNAs	Known by sequence	13	12
	Known by structure	10	16
	Previously predicted	143	52
	Novel predicted	549	260
	
	Total	715	340

UTR elements	Known by sequence	24	2
	Known by structure	44	16
	Previously predicted	69	6
	Novel predicted	174	27
	
	Total	311	51

Sum of all RNA structures		1,026	391

Table 1: Overview of all conreads in the PigEST data consisting known and putative novel ncRNAs and cis-acting (UTR) regulatory RNA elements. Known RNA families were found through sequence similarity to ncRNA databases and structure similarity to covariance models of Rfam, whereas tRNA candidates are not counted due to a high rate of tRNA pseudogene annotations [see Additional file 1, Table S11 for a similar table inluding the tRNA candidates]. Previously predicted RNA secondary structures overlap with greater than 50% a RNAz or Evofold prediction in human reported in [16] and [17]. Novel predictions comprise the remaining high confidence RNAz candidates. Cis-acting (UTR) elements are predicted RNA structures conserved in an human UTR (no matter if conreads contain an ORF) and ncRNAs represent conserved RNA structures predicted in ORF-free conreads without similarity to annotated UTRs.

The PigEST sequences contain 18,621 protein-coding RNAs which were detected directly by sequence similarity to protein databases, see [32]. Protein-coding mRNAs are expressed mostly in large quantities, hence 80% of them form contigs. With the exception of SRA1 [34] there are no known structured RNAs within ORFs, hence we removed the ORF regions. Nevertheless, the coding conreads were searched for cis-acting structured RNA elements in up- and downstream UTRs. The remaining ORF-free conreads are potential candidates for ncRNAs as well as UTR elements.

### Homology search in mammals and few more distant species

The similarity search revealed that 27,578 contigs and 18,033 singeltons are at least partially but uniquely conserved on 71,112 loci in the cow (see methods), so far the most closely related mammal for which a genome has been sequenced. The conserved conreads consist of one-third of porcine ORF-free conreads (15,374 contigs, 15,552 singletons) and 80 percent of the protein-coding transcripts (12,204 contigs and 2,481 singletons). The remaining 76,189 conreads have no homologous sequence in the cow genome, hence they could not be further analysed by comparative genomics. This large amount of unaligned assembled ESTs could represent low quality singletons, transcriptional background or pig specific transcripts. At least in part, this large number is most likely an artifact since the current assembly of the bovine genome does not cover the entire genome [35]. For 15,773 pig conreads, we observe split alignments mapping to different loci on the cow genome [see Additional file 1, Figure S3 for the number of loci per conread]. 77% of these map to the same chromosome in the same reading direction as one would expect for regularly spliced transcripts [see Additional file 1, Table S3]. The remaining cases are either EST sequencing artifacts, assembly problems in the current release of the cow genome or in the PigESTs. Conceivably some of them are exceptional transcripts such as the ones described in [31].

We then searched for similarities of the pig conreads with the 16 other vertebrates aligned to cow in the UCSC Genome Browser [36]. We started from the 71,112 cow loci that we previously identified as homologous to a pig conread and considered both the pairwise blastz alignments [37] and the multiz alignments [see Additional file 1, Table S4] provided through the Genome Browser [38]. We found for 66,350 loci a similar sequence in at least one additional species. The investigation of cow-human (65,196 homologous loci) and cow-mouse (55,416 homologous loci) pairwise alignments revealed that significantly more PigEST orthologs exist to human. This agrees with the already published thesis that the pig sequence space is closer to human than mouse [39]. In the 4,227 pig-cow specific mappings there are only 177 protein-coding conreads (264 loci) included, which supports the lower evolutionary pressure of ORFs as well as their higher conservation. The mappings can be accessed online via the PigEST website [40].

### Known non-coding RNAs and cis-acting RNA elements

**Figure 2:**
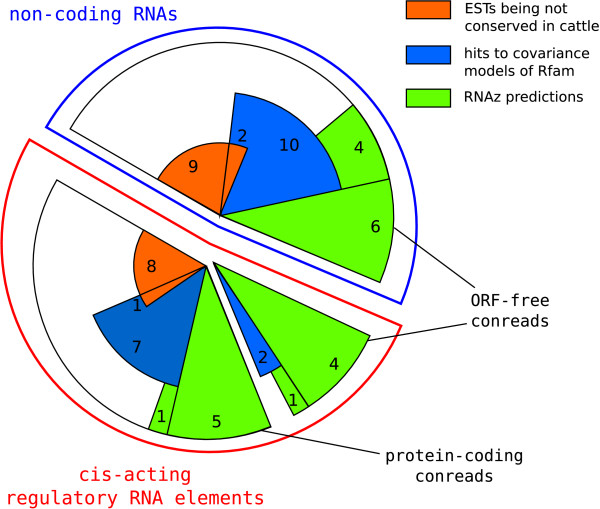
Known ncRNAs in the PigEST data, which were detected by sequence similarity to ncRNA databases, are ordered according to their pipeline results. Of these 51 conreads, whereas tRNA hits are not considered, 25 conreads are located far away from any known gene. These are labelled as ncRNAs. In 20 conreads with a known ncRNA is also an ORF located, of which 12 are approved by the detection of a conserved RNA structure as cis-acting regulatory RNA elements. Additional six ORF-free conreads have an RNA structure conserved in a human UTR. Pig conreads, which are not conserved in at least the closely related mammal cow, are not applicable for comparative computational approaches.

We annotated 13 contigs and 12 singletons as functional ncRNAs by simple sequence similarity to the ncRNA databases Rfam [41], RNAdb [42], fantom3_noncoding [43] and miRBase [44] [see Additional file 1, Table S2]. This set includes 14 miRNAs, 2 snoRNAs and 2 rRNAs. Sequence similarity to common cis-acting regulatory RNAs was found in 24 contigs and 2 singletons, of which 20 conreads contain an ORF not overlapping the RNA database matches. These annotations without the tRNAs had already been done in [32]. See Figure 2 for an overview of sequential known functional RNA structures in pig and there classification in the further pipeline. In addition, 19 tRNAs are located in ORF-free conreads (12 singletons) and 42 tRNAs in protein coding conreads (39 singletons).

The RaveNnA [45] scan using covariance models of known RNA structures revealed additional 54 contigs and 32 singletons matching 33 structures in Rfam v7.0 [41] [see Additional file 1, Table S2). Of these, 44 contigs and 16 singletons are cis-acting RNA elements, of which 34 known RNA structures are located in protein-coding transcripts and 26 in ORF-free conreads. Again, the most frequently detected ncRNAs are microRNAs, snoRNAs and snRNAs. The total list of known cis-acting regulatory elements contains 37 Selenocysteine insertion sequences (SECIS), 15 Histone 3'-UTR stem-loops, and 8 Iron response elements. Additional tRNAs are detected in 34 contigs and 67 singletons through structure similarity. Approximately 100 tRNA candidates were successfully verified by tRNAscan_SE [46]. Half of these were predicted as pseudogenes which are unusual tRNA homologues. Some functional ncRNAs derive from tRNAs. The most prominent example is the BC1 transcript in rodents [47], other tRNA/SINE-derived functional ncRNAs were recently described in [48]. Our collection of expressed tRNA pseudogenes thus could contain novel tRNA-derived functional ncRNAs.

In summary, we obtain 137 known ncRNAs and cis-acting RNA elements by sequence similarity and structure similarity excluding tRNA candidates (see Table 1).

### Candidates of novel non-coding RNAs and cis-acting (UTR) RNA elements

**Figure 3:**
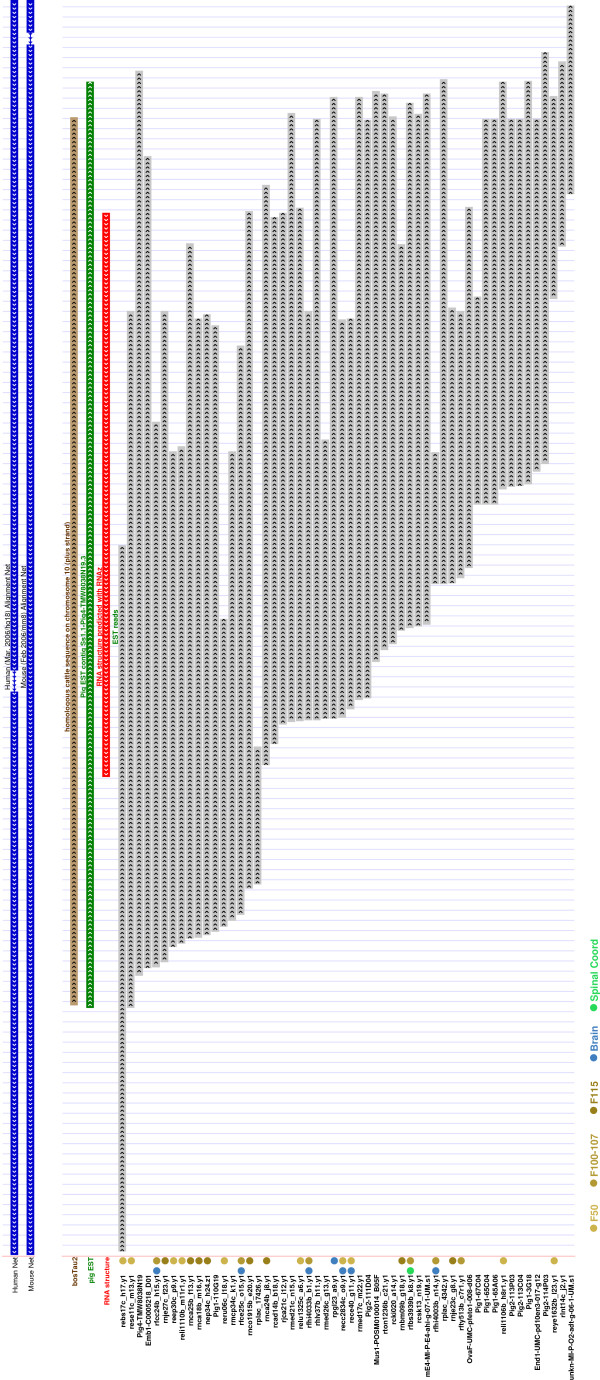
Genome browser snapshot of the pig contig *Ss1.1-Pig4-TMW8038N19.3 *and its constituent sequences [36, 74]. The contig is assembled from 55 overlapping reads. It is conserved in the cow chromosome 10 with 95% of its 780 nucleotides as well as the minus strand of human chromosome 14 and mouse chromosome 14. An RNA structure was predicted with high confidence by RNAz covering 61% of the entire contig. The contig is expressed in 29 distinct cDNA libraries. The read expression in selected tissues and their developmental stages are shown. F50, F100-107, F115 indicates the different foetal stages, F115 being the new born pig, 115 days after breeding.

**Figure 4:**
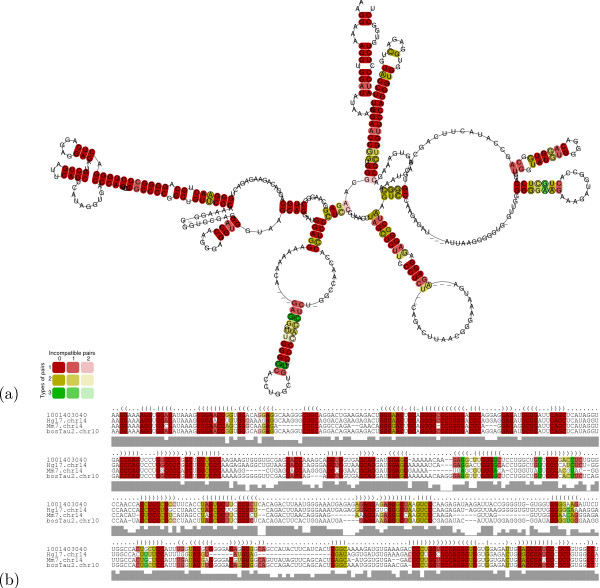
The high confidence RNA structure of the pig contig *Ss1.1-Pig4-TMW8038N19.3 *from Figure 3 is shown. (a) RNA structure calculated by RNAalifold [75]; (b) Alignment of the pig, human, cow, and mouse with annotation of the conserved structure.

**Figure 5:**
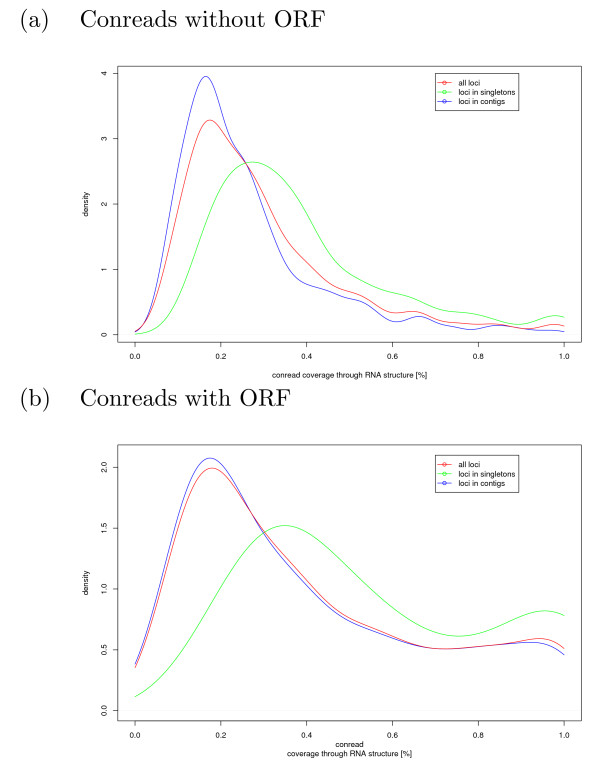
Conread coverage through conserved RNA structures predicted as high confident by RNAz is illustrated for (a) conreads without an ORF and (b) conreads containing an ORF. The density is computed with a gaussian kernel by R [76].

The analysis of conserved pig transcripts by RNAz [11] predicts a high confidence secondary RNA structure in 1,795 conreads (thereof 1,412 contigs) [see Additional file 1, Table S5]. More than two-thirds of the predictions in ORF-free conreads are contigs (825 in contrast to 338 singlereads), even though the input consists of a larger amount of singletons. About 60% of all predicted RNA structures are conserved in cow, human and mouse [see Additional file 1, Table S6 for ORF-free conreads]. A representative contig with its predicted structure is shown in Figure 3 and 4. The length of the predicted structure elements in ORF-free conreads is 143 ± 56 nt, close to the window size of 120 nt used by the RNAz program. The conread coverage rate by predicted RNA structures is shown in Figure 5, whereas the average structure coverage of coding transcripts is larger (see Figure 5(b)). With slightly more relaxed RNAz criteria (*p *> 0.5), approximately 19,300 conreads have conserved secondary structures.

A control screen of the RNAz predictions using shuffled alignments as described in [16] yields an estimated False Discovery Rate (see methods for FDR calculation) of about 10% for ORF-free conreads. The CONC program, which uses an SVM to discriminate ORFs from other transcripts [49], did not provide additional information: CONC identified all RNAz predictions in ORF-free conreads as ncRNAs, which strengthens the rejection of RNAs with open reading frames (ORF) through the pipeline.

#### Reading direction of structured RNAs

The machine learning tool RNAstrand [50] classified 861 RNA structures to exist on the reverse complementary strand of non-protein coding conreads. In contrast, only 388 ones have a larger evidence to exist on the positive conread reading direction [see Additional file 1, Table S7]. A similar proportion of reading directions was observed for predicted RNA structures in coding conreads.

#### Mapping of structured RNAs to annotated UTRs

Predicted RNA structures, which are similar to known UTR regions, are counted as cis-acting (UTR) elements. Therefore, the more likely reading directions of RNAz predicted loci were scanned against the known gene annotation of human (hg17, May 2004) [51].

Of the RNA structures predicted in ORF-free conreads, 86% are conserved in human. We observed that 85% of the RNAz predictions are located far away from any known human gene (and UTR). These 1,004 conreads are labelled as putative ncRNAs in the porcine transcriptome. The remaining 15% (177 conreads) are homologous to human UTRs. Here, they are considered as putative cis-acting (UTR) RNA elements [see Additional file 1, Table S8]. Of these, 18 conreads contain a second RNA structure which is not similar to a human UTR and therefore they are also counted as ncRNA. The putative cis-regulatory (UTR) elements are located mostly on the sense strand, whereby the ratio of predicted ncRNAs on sense conreads is decreased to 29%. However, more than 35% of human conserved secondary RNA structures in pig are aligned to the reverse complementary strand of an annotated human UTR.

In addition to these we also investigated all the coding conreads, and we predicted high confidence RNA structures in 99 of these (95 contigs, 4 singletons), which do not overlap codon sequences, but are similar to a human UTR [see Additional file 1, Table S8]. These are around 40% (82 loci) of sense RNA structures as well as only 3% (18 loci) of antisense RNA structures of the human conserved coding conreads, which comprise 84% of all RNA structures in coding conreads. They are labelled as putative UTR elements together with the 177 ORF-free conreads described above. In total, 82% of the cis-acting (UTR) RNA structures are predicted in the positive conread reading direction. All high confidence putative ncRNAs and cis-acting RNA elements are available as the PigEST-ncRNA webserver [52].

The other human conserved RNA structures predicted on the positive strand of coding conreads are putative unspliced intronic ncRNAs or false positives. The predictions in the negative reading direction could be further candidates of independent transcriptional units. With slightly more relaxed RNAz criteria, the corresponding numbers are roughly 1,800 ORF-free conreads and 1,500 protein-coding transcripts which comprise predicted RNA structures in regions similar to known human UTRs, as well as around 9,700 ORF-free conreads with putative ncRNAs.

To summarise, high confidence novel RNA structures are predicted in 1,262 conreads. Together with 137 known RNA structures, the PigEST resource comprises trans-acting elements (ncRNAs) in 715 contigs and 340 singletons as well as cis-acting elements (inside UTRs) in 311 contigs and 51 singletons, of which 18 conreads contain both predictions of trans- and cis-acting elements (see Table 1).

#### MicroRNAs

**Figure 6:**
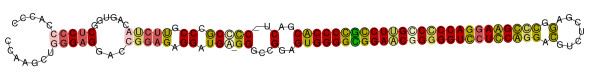
For the contig *Ss1.1-rese12c_n15.5* we predicted three non-overlapping   conserved secondary RNA structures. Inside one novel RNA structure prediction,   we found a conserved microRNA structure by RNAmicro at the conread positions 87   to 220 in the positive reading direction with *p* = 1, which is   illustrated in Figure 6. The alignment to cow, human chromosome 11 (3'UTR of   hypothetical protein *NM_001007139* (GenBank)) and mouse has an average sequence   identity of 71.5%. The structure was calculated by RNAalifold and colored by colorrna.pl, being both from the Vienna RNA Package [75].

Using RNAmicro [53] (classifier *p *> 0.9) on the ORF-free conreads having an RNAz match, we obtained miRNA predictions for 95 contigs and 32 singletons. Together with known miRNAs, these cover in total 143 loci. As for RNAz screens, we estimated that the False Discovery Rate of RNAmicro screen is approximately 11%. Further details can be found in Additional file 1, Table S9. It should be noted, however, that 22 candidates aligns to human UTRs and should therefore be treated with caution. On the other hand, one of the known human mir-196 paralogs is located in the 5'UTR of a *HoxA9 *transcript [54]. A putative novel miRNA structure in the contig *Ss1.1-rese12c_n15.5 *is depicted in Figure 6 as an example.

#### Comparison with other screens for structured RNAs

Of the RNA structures which are conserved in human, 22% (199 loci) of the ncRNA candidates and 27% (77 loci) of UTR elements overlap (coverage >50%) a prediction from [16] or [17]. These previously predicted structures occur in 270 conreads. On the other hand, the porcine transcriptome comprises 809 conreads with predicted ncRNAs located far away from a human ORF and 201 conreads with a cis-acting element which have not been reported before.

#### Transcriptional evidence in other organisms

To check for transcriptional evidence in other organisms, we searched in the NCBI dbEST [55] database and obtained a huge amount of hits. Similarities have been noticed in 73% of the RNAz predictions to ESTs discovered in other organisms than pig. High significant hits with an identity >95% and RNA structure coverage >80% have been detected for 30% of the conreads.

### RNA structure predictions in known functional RNAs

The sequences which were matched initially by sequence and/or structure similarity to known functional RNAs listed in ncRNA databases, also are passed through the rest of the pipeline as a control. RNA structures are predicted by RNAz in six microRNAs and four cis-regulatory elements. The latter are confirmed through their homology to human UTR regions [see Additional file 1, Table S10]. In eight cases the genomic location of the known RNA and the prediction coincide. With more relaxed blastn options, described in the methods part, we found five additional annotated RNA structures (3 snoRNAs, 2 cis-acting elements) which were predicted by RNAz. Nevertheless, there are 16 conreads being detected as functional RNAs due to sequence similarity, which are not conserved in cow (Figure 2) and could not be verified by comparative genomic approaches.

### Mapping the ncRNA candidates onto the PigEST cDNA Libraries

Using the expression information data from the PigEST resource [32] we inferred expression of the 715 contigs and 340 singletons containing ncRNA predictions or matches against Rfam (by sequence or structure similarity), where tRNA predictions were discarded due to apparent pseudogene annotation in the set. We conducted expression analysis of the corresponding conreads containing ncRNA predictions. The PigEST resource contains in total 92 useful non-normalized cDNA libraries from which expression patterns can be extracted. The expression of a contig in a cDNA library is simply counted as the fraction of EST reads from that library which are assembled into the contig.

**Figure 7:**
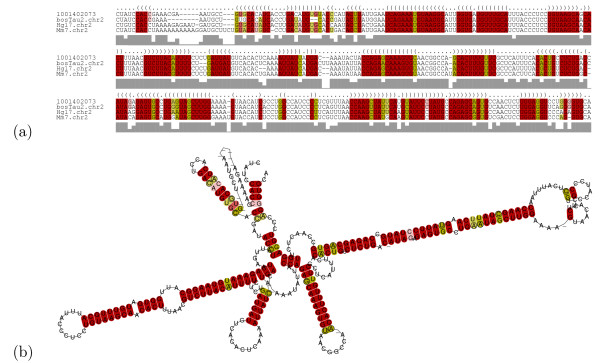
The contig *Ss1.1-Mixc-0038,l13.5 *is expressed in 14 cDNA libraries. It contains an RNA structure already predicted in [16] covering 32% of the transcript. (a) Alignment of the pig, cow, human and mouse with annotation of the conserved structure; (b) RNA structure calculated by RNAalifold [75].

Using this we found that there are 114 such contigs with expression in at least 10 cDNA libraries (see one representative contig in Figure 3 and 4). If we require that at least two reads must be present in all libraries, this number reduces to 24 [see Additional file 2]. Note that additional public EST reads can be present as well. Of these only 5 (*Ss1.1-rsug22 m15.5, Ss1.1-Mixc-0038,l13.5, Ss1.1-Pig4-TMW8032L01.3, Ss1.1-rese12c l8.5 *and *Ss1.1-Pig4-TMW8061A10.3*) contain prediction known from previous scans in human. On the other hand, 3 contigs are only conserved in cow. The conserved RNA structure in *Ss1.1-Mixc-0038,l13.5*, which has the longest conread coverage of all these candidates with 32%, is presented in Figure 7.

**Figure 8:**
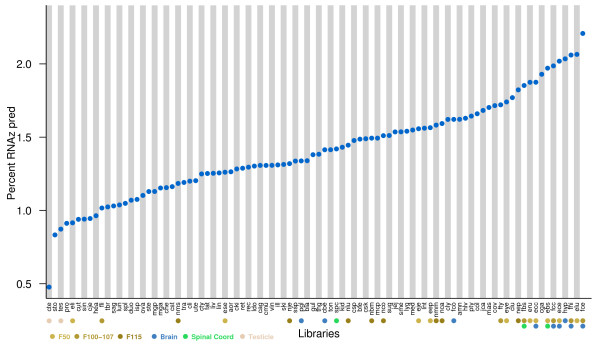
For each cDNA library the fraction of conreads containing a ncRNA candidate sequence or structure match to Rfam or a high confidence ncRNA prediction, is indicated. Selected tissues and their developmental stages. F50, F100-107, F115 indicates the different foetal stages, F115 being the new born pig, 115 days after breeding.

The fraction of conreads containing ncRNA candidates in each cDNA library is on the order of 1–2% (Figure 8). Intriguingly, we see that developmental and neuronal related tissues in general contain a higher level of conreads with ncRNA predictions. It is also remarkable that testes contain a small fraction taking into consideration that testes, brain and developmental tissues are among the most diverse tissues, that is containing most different expressed genes [32]. 

## Discussion

We have implemented a pipeline to detect known and novel evolutionary conserved ncRNAs in assembled EST data through a combination of several stand-alone bioinformatic tools. As well as making ncRNA predictions, it also detects protein-coding RNAs and cis-acting regulatory regions (in UTRs) of mRNAs. We detected a large number of evolutionary conserved thermodynamically stable RNA structures in both ORF-free and protein-coding conreads. These conreads are plausible candidates for novel mRNA-like (polyadenylated) ncRNAs, many of which are spliced. The candidate set does not contain, on the other hand, large amount of intronic, poorly conserved, or non-structured ncRNA.

**Figure 9:**
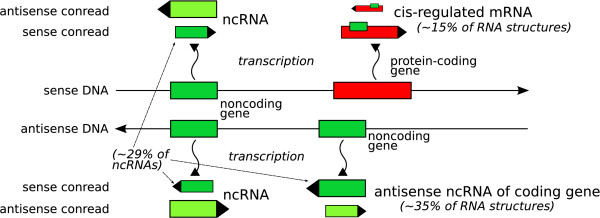
The ncRNA candidates in the PigEST data are predicted in different locations relatively to the protein-coding genes. The size of the transcripts in the figure approximately indicates their observed amount.

Surprisingly, the EST data – as in other EST projects – also contain a large number of housekeeping RNAs which are not normally polyadenylated. These are either technical artifacts of the cloning procedure or, more likely, they indicate that ncRNAs polyadenylation is a common phenomenon throughout eukaryotes. In either case the data show that EST projects provide a valuable source of ncRNA sequences. Based on the matches to the cow genome, we infer that the predicted RNA structures presumably are distributed almost evenly across the entire pig genome (see Figure 9). One-third of the predicted conserved RNA structures in the PigEST resource are located in antisense direction relative to the reading direction of coding gene UTRs. One explanation is that we see at least in part expressed (retro)pseudogenes that regulate genes through hybridization [4,56]. The large amount of RNA structures predicted in the reverse complementary conreads can be explained by the possible existence of overlapping sense-antisense transcripts, an apparently very common phenomenon in mammals [57-59].

Protein-coding mRNAs are expressed on average at a much higher level compared to non-coding transcripts. As a consequence we observe that mRNAs, in contrast to ORF-free ESTs, are predominantly located in contigs rather than singletons. A high level of conservation of protein-coding sequences between pig conreads and the bovine genome emphasizes the similarities between the mammalian mRNA complements. In contrast, a higher rate of ncRNAs was predicted in singletons. These ncRNAs are probably expressed at low level which is also observed through the mapping to the individual PigEST cDNA libraries. The sequence conservation with other species is also much less pronounced for the ORF-free conreads. These observations are consistent with the idea that the non-coding parts of the transcriptome are much more variable between tissues and species [56].

In our data, the predicted RNA structures can be associated with protein coding genes either because the RNAz signal is located on a protein-coding conread or because an ORF-free conread shows significant sequence homology with a human UTR. The fraction of RNA structures in both groups is almost the same, hence they are biologically indistinguishable. One possible reason for the high abundance of UTR conreads that do not also contain ORFs is that UTRs with extensive secondary structures are larger and/or have a higher probability to give rise to truncated ESTs.

The pipeline could further be improved by including methods such as CMfinder [60] which can search for RNA structures in multiple sequences with low sequence similarity. Such an approach has shown to supplement methods like RNAz when the sequence similarity is so low that it affects the quality of multiple alignments made from methods based solely on sequence similarity (Torarinsson et al: Comparative genomics beyond sequence based alignments: RNA structures in the ENCODE regions, submitted). The expression analysis of the ncRNA candidates shows that most of these transcripts are cell-type specific. This observation might in part be due to the insufficifent cDNA library sizes. However, we obtained a four fold higher number of predictions in contigs than in singletons, supporting the cell specifity. In further contrast, more than 100 ncRNA candidates are expressed in at least ten cDNA libraries indicating transcription beyond the noise level. Strikingly the putative developmental regulatory ncRNAs are expressed at the highest level, which in agreement with earlier genomic analyses in other mammals [22,61,62]. Despite the strong computational confidence, only laboratory work can give a final verification.

## Conclusion

In conclusion, the computational screen of the PigEST resource reported here provides strong confidence for a large number of conserved secondary structure elements in polyadenylated transcribed RNAs. The low expression levels of the predicted RNA candidates together with the observations that a larger relative fraction of them is found in cDNA libraries from developmental stages is in good agreement with the current paradigm of ncRNA roles in higher organisms and supports the thesis of functional polyadenylated ncRNAs. Since these seem to appear in low number in developmental tissues, this in itself provide a plausible explanation of why they previously have been overlooked.

## Methods

The EST2ncRNA pipeline, presented in Figure 1, predicts ncRNA candidates by removal of all ORF regions from the source conreads. The pipeline compares all conreads to a closely related species for which a sequence is available, in this case the cow. This genome is then used as reference for homology searches in further organisms and for the construction of multiple alignments. These are screened in the next step for evolutionary conserved and thermodynamically stable structured ncRNAs using RNAz [11]. At this stage, another algorithm could readily substitute RNAz. Predicted RNA structures that can be aligned with an UTR of another organism are labelled as "cis-regulatory" (UTR) elements. RNA structures in protein-coding conreads, which are not similar to an annotated UTR, are not further considered.

### Data from the PigEST resource

The PigEST resource is based on 1,021,891 porcine EST sequences from which 636,516 were extracted from the Sino-Danish PigEST resource [32] and 385,375 from GenBank [63]. The Sino-Danish PigEST resource originates from one normalized and 97 non-normalized cDNA libraries representing 35 different tissues and three developmental stages. The sequences were assembled by distiller [64] resulting in 48.629 contigs and 73,171 singletons. Protein-coding RNAs were searched by sequence similarity to the protein databases NCBI nonredundant and UniProt [65]. As pragmatic selection criteria were applied an identity > 60% and known protein sequence coverage > 50% [32].

### Sequence and structure similarity to known ncRNA families

The following ncRNA databases were scanned for primary sequence and secondary structure similarities: RNAdb (August 2004), Rfam (release 7.0, March 2005), miRBase (release 8.1, May 2006), fantom3_noncoding (release 3.0). The local blastn [66] searches were performed with standard parameters and selection thresholds of E-value < 10^-20^, identity >95% and subject coverage >85%.

The Rfam 7.0 covariance models, which represent known ncRNA families, were scanned by RaveNnA [45] to find structural similarity. The parameters (local or global alignment, window length) for each Rfam model were taken from the annotation file of the Rfam seed alignments [67]. Since RaveNnA produce many low score hits, the candidates were further filtered. Hits were therefore ignored if (i) the matched conread regions included gaps, (ii) the modelled subsequences were shorter than 60% of the model window length or (iii) the number of modelled basepairs was not in a range of 20% around the basepair number of the model. Transfer RNAs were searched by tRNAscan_SE version 1.23 with default parameters, which combines tRNA covariance models with several heuristics.

Functional RNA elements in protein-coding conreads not overlapping the coding sequence were considered as cis-acting (UTR) RNA elements in the subsequent analysis.

### Homology search

The source pool was mapped against the genome of a closely related organism. The closest pig related even-toed ungulates (Artiodactyla) with an existing genomic sequence project [35] is the bovine *Bos taurus *(Btau_-_2.0, June 2005). Two parameter settings of blastn were applied for aligning EST data to genomic data, which were the standard parameter and EST specific parameter allocation recommended for annotation of genomic DNA with ESTs [68]. The latter variant was realised through an adapted serial blast strategy [69] consisting of three steps to overcome the time problem: (i) a first blastn search with standard parameters, (ii) aligned conreads were retrieved, (iii) a second blastn search with EST specific parameters for the retrieved conreads against their related chromosomes. The result part is based on the blastn hits with EST specific parameter allocation. The non-overlapping blast hits with lowest E-value were filtered as conserved conreads if their E-value was less than 10^-20^, their length greater than 100 nt and a 100% identical sequence of at least a length of 75 nt exist. Conreads, which were not conserved in the closely related organism, were rejected from the pipeline and were stored as putative source organism specific transcript candidates.

More similar sequences to the conserved conreads in other organisms were detected by available genome-wide pairwise or multiple alignments from the UCSC genome browser [38] of an organism which was already aligned to the assembled ESTs. The pairwise alignments of cow (bosTau2, Mar. 2005, Baylor Btau 2.0) to human (hg17, May 2004) and mouse (mm7, Aug. 2005, NCBI Build 35) were available as chain files and were scanned by the UCSC liftOver tool [70] with a conread coverage ratio of 0.8. This implies that at least 80% of the reference organism subsequence had to match the alignment. The multiple alignment of human (hg17) to 11 mammals, three actinopterygii, one amphibia and one aves, generated with multiz, was available as maf file and was scanned with a conread coverage ratio of 0.6. The applied ratio was smaller for multiple alignments due to typically shorter alignments. To obtain multiple alignments containing the porcine conreads, we realigned the pig sequences to corresponding UCSC alignments by clustalw [71].

### Prediction of conserved stable secondary structures

The alignments had to be pre-processed before RNAz 1.0(pre-release) [11] could be used to search for thermodynamically stable and conserved secondary RNA structures. In addition to several cleaning steps, which are described in the RNAz manual [72], the rnazWindows.pl tool sliced the alignments in 40 nt overlapping windows of size 120. This allows RNAz to find local structures. The pre-processed alignments were scored with RNAz using standard parameters plus the *-both-strands *parameter to score also the reverse complementary alignments. All alignments with classification score *p *> 0.5 were stored as conserved secondary RNA structures. The overlapping windows in the positive RNAz predictions were combined into clusters (loci) without attention for their strand predictions by the rnazCluster.pl script, which is also part of the RNAz package. RNAz predictions with RNAz classificator *p *> 0.9 and high thermodynamical stability described by a z-score < -3 were interpreted as high confidence hits.

The confidence of the predictions was measured by running RNAz again with randomized alignments. Therefore, the positions in the alignments of the preprocessed maf files were mononucleotidely shuffled with the RNAz tool rnazRandomizedAln.pl. The program aims to remove any correlation arising from a natural secondary structure while preserving mean pairwise identity and base composition. The false discovery rate was calculated as number of random hits related to native hits.

A locus was counted as previous ncRNA prediction if it overlaps a prediction of the RNAz screen [16] or the EvoFold screen [17] of the human genome with a subject coverage of greater than 50%.

### EST specific blastn parameters

The standard parameters of blastn are optimized for short alignments with a high identity and a short execution time. However, we were interested in a high conread coverage during the search for conread homologous genome sequences. Thereby, a small decrease of identity is justified due to the low quality of EST sequences and the structure conserved mutations in ncRNAs. The standard parameter allocation was compared with the one recommended for noncoding queries [73] and the one recommended for genomic DNA annotation with ESTs. The best balance between expected alignment length and percent identity, which are calculated by the High-scoring Segment Pair (HSP) of the blast algorithm, was provided by the EST specific parameters [see Additional file 1, Table S1].

The alignments of the PigEST data to the cow genomic data were provided by EST specific blastn parameters and standard parameters. Alignments generated with EST specific parameters were generally longer [see Additional file 1, Figure S1], which is more appropriate to find homologs to entire RNA transcripts. In general, EST specific alignments resulted in more positive RNAz predictions and the most structural RNA elements were predicted from both alignment sets [see Additional file 1, Figure S2]. Nevertheless, one third of positive predictions based on standard alignments were not detected by the other variant. These ESTs include possibly highly conserved short structures.

### NcRNA candidates as cis-regulatory elements

The more reliable conread strand of each predicted conserved RNA structure (locus) was identified by RNAstrand v1.1.0 [50], which, like RNAz, RNAmicro and CONC, is based on a support vector machine (SVM) classifier. The prepared alignments of the related windows of each locus were merged and applied as input to the already trained SVM. The human gene annotation was used to identify RNA structures conserved in UTRs. RNAz hits are putative cis-acting elements, if their homologous human sequences overlap the UTRs of known genes, which are annotated in the *knownGenes *table (hg17, May 2004) of the UCSC genome browser [38]. As mapping criteria was used an overlap of at least one base of the human homologs to a human UTR, whereas the RNAstrand predicted locus strand was applied. By cis-acting element candidates we considered only those matching known UTR regions, even though they can also occur outside of UTRs.

### Further methods

Putative novel miRNA precursors were detected by RNAmicro v1.1 [53] in both reading directions of the alignments of positive RNAz predictions with window sizes 70, 100 and 130 nt and step size of 5 nt. RNAmicro assumes that input alignments include consensus secondary structures, wherefore the alignments of positive RNAz predictions were used. P-values > 0.9 were stored as putative microRNAs, whereas overlapping windows on the same strand were combined. Like RNAz results, RNAmicro predictions were verified by shuffled input alignments.

CONC v1.0 [49], a tool that predicts whether a sequence is protein-coding or not, was applied on all RNAz predicted loci of ORF-free conreads to verify them. To this end all 6 reading frames were investigated. Additional sequence similarities of the predicted RNA structures to known ncRNAs in the ncRNA databases were searched by blastn with more relaxed criteria, being an identity > 85% and a subject coverage > 60%. Furthermore, the high confidence ncRNA predictions were compared with the NCBI dbEST [55] database by standard blastn parameters and E-value < 10^-10^.

## Availability and requirements

Perl source code of the 'EST2ncRNA' framework and the corresponding documentation are available for download from . At the same website we also provide the predicted ncRNAs and cis-regulatory elements. The comparion of Porcine and Bovine ESTs can be found at .

## Authors' contributions

PFS, ILH and JG formulated the project. SES implemented the pipeline and applied the pipeline on PigEST data. JG generated the expression analysis. All authors contributed to and approved the final manuscript.

## Supplementary Material

Additional file 1 Supplementary tables and figures. (A) Comparison of different blastn parameter allocations in the PigEST-cow alignments and (B) additional tables and figures reporting further results of the pipeline. Click here for file

Additional file 2 Widely expressed ncRNA candidates. A list of ncRNA candidates expressed in at least 10 cDNA libraries with at least two EST reads in each. Click here for file
