# Supplementary material for: Detection of RNA structures in porcine EST data and related mammals
Source: BMC Genomics. 2007 Sep 10;8:316. doi: 10.1186/1471-2164-8-316 (PMC2072958; doi:10.1186/1471-2164-8-316)
Supplement: Additional file 1 — Supplementary tables and figures. (A) Comparison of different blastn parameter allocations in the PigEST-cow alignments and (B) additional tables and figures reporting further results of the pipeline. [file 1471-2164-8-316-S1.pdf]

Additional File 2
of
"Detection of RNA structures in porcine EST data and related mammals"

July 28, 2007

Blastn parameter allocation for pigEST–cow alignment

Comparison of three parameter allocations of blastn		
parameter allocation	length	identity
-r 5 -q -4 -W 7 -G 10 -E 6	64.538 nt	65%
-r 1 -q -1 -W 9 -G 1 -E 2	43.175 nt	75%
-r 1 -q -3 -W 11 -G 5 -E 2	18.723 nt	99%

Table S1: Three parameter allocations of blastn are compared by the expected HSP length and the expected percent identity. The first raw represents the parameter for noncoding queries, the second one for EST specific queries and the third one the standard allocation. The parameters r and q describe the reward as well as the penalty for matches and mismatches. W is the word size. G is the cost to open a gap and E is the cost to extend a gap.

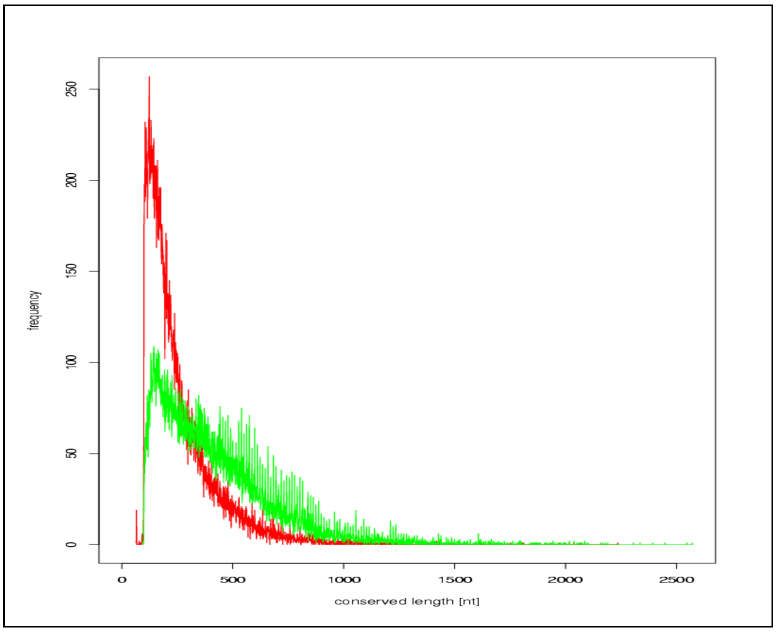

Figure S1: The length distribution of local alignments between pig ESTs and cow genome shows that blastn with EST specific parameters generates in general longer alignments. These ones are more appropriate to find homologs to entire transcribed RNAs. The green curve presents blastn with EST specific parameter allocation and the red curve presents blastn with standard parameters.

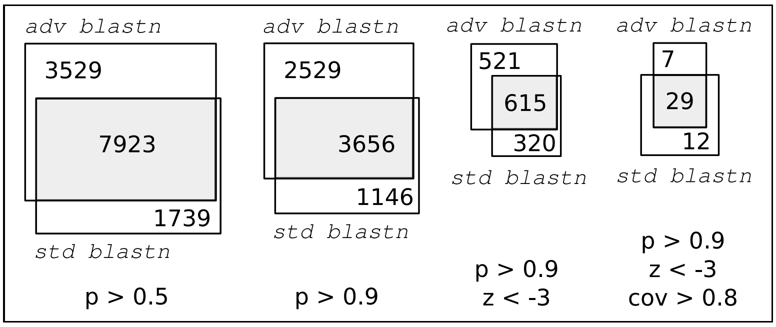

Figure S2: The commonalities of positive RNAz predictions, based on two parameter allocations of the pig-cow alignment, are illustrated as Venn diagrams for different classification criteria. In general, in EST specific alignments (labeled as 'adv blastn') are more conserved RNA structures predicted as in alignments generated by blastn with standard parameters. The most candidates originate from both variants.

Additional tables and figures reporting further results of the pipeline applied on the PigEST data

Known ncRNAs and cis-acting RNA elements			
	ncRNA	cis-acting RNA	total
Blast (sequence similarity)	25 (44)	26 (68)	51 (112)
RaveNnA (structure similarity)	39 (150)	69 (104)	108 (254)
total	51 (172)	86 (130)	

Table S2: Several known ncRNAs (trans-acting) as well as cis-acting RNAs are identified in the pigEST data through sequence similarity to ncRNA databases and structure similarity to covariance models of Rfam. The tRNA candidates are considered only in brackets due to a high rate of tRNA pseudogene annotations. The detected RNA family determines the classification as ncRNA or cis-acting regulatory RNA element. However, known sequences which are located in protein-coding conreads are always considered as cis-acting elements. This set includes 42 tRNAs, but also one snoRNA. All known functional sequences are reviewed through RNA structure prediction if the EST was aligned at least to cattle.

PigEST conservation in the cow genome		
	ORF-free conread	coding conread
total number	30,926	14,685
one locus, one chromosome	25,409	4,429
>1 loci, one chromosome, one strand	4,834	7,358
>1 loci, one chromosome, both strands	46	220
>1 loci, >1 chromosomes	637	2,678

Table S3: The PigEST conservation in the bovine genome (bosTau2) was found by blastn with EST specific parameter allocation (see Table S1). Beside the normal case of one or more conread substructures conserved on one cow chromosome on the same strand, there exist also several alignment artefacts, which are numerated here.

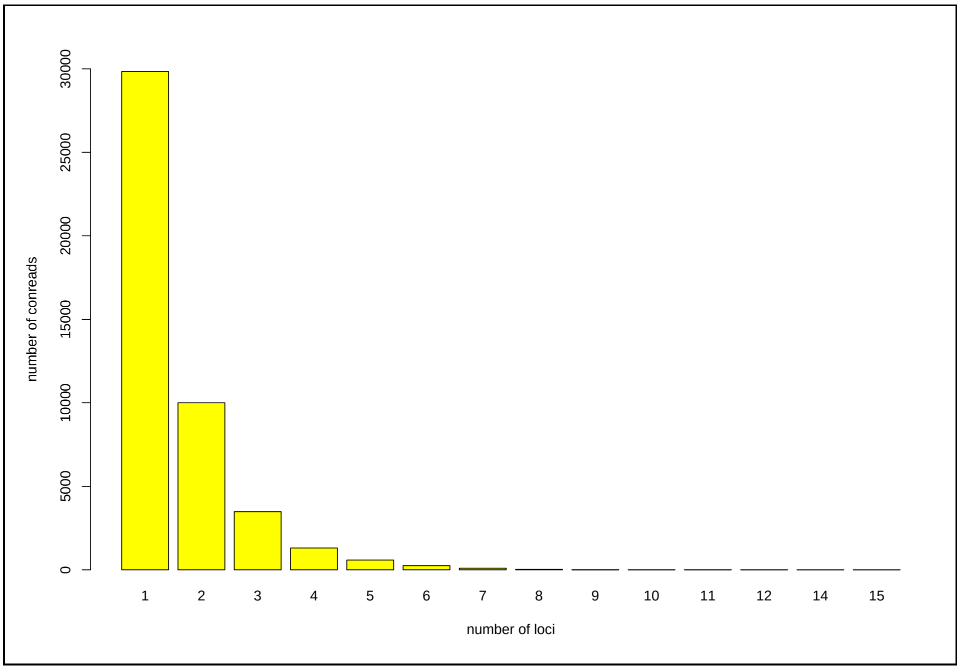

Figure S3: The pigEST–cow alignment found 45,622 at least partially conserved conreads with similarity in 71,112 non-overlapping loci by blastn with EST specific parameter allocation. The number of loci per conread is illustrated in a barplot.

PigEST conservation using UCSC human multiple alignments		
aligned organism	ORF-free conread	coding conread
human (hg17)	7,847	8,567
chimp (panTro1)	7,661	8,423
dog (canFam2)	7,627	8,493
macaque (rheMac2)	7,628	8,422
mouse (mm7)	7,172	8,484
rat (rn3)	7,040	8,369
elephant (loxAfr1)	6,107	7,551
rabbit (oryCun1)	6,098	7,665
tenrec (echTel1)	5,944	7,827
armadillo (dasNov1)	5,822	7,415
opossum (monDom2)	5,423	7,803
chicken (galGal2)	3,106	6,151
frog (xenTro1)	2,591	6,065
zebrafish(danRer3)	2,342	6,205
fugu (fr1)	2,335	6,230
tetraodon (tetNig1)	2,260	6,290

Table S4: The PigEST conservation in 11 mammals (over the first line), 1 aves, 1 amphibia and 3 actinopterygii is presented for ORF-free and protein-coding conreads. The latter are more conserved, at least in the very distant organisms. The data is extracted from the multiple alignment of human to 16 other species offered by UCSC genome browser. Thereby, the cow serves as reference organism to the pig conreads.

Conserved RNA structures in pig conreads predicted by RNAz								
			ORF-free conread			coding conread		
p-value	z-score	coverage	Contigs	Singletons	loci	Contigs	Singletons	loci
< 0.50	–	–	26,849	64,878	–	8,036	2,727	–
> 0.50	–	–	6,871	4,581	15,534	6,873	985	11,705
> 0.90	–	–	3,956	2,229	7,465	3,336	362	4,436
> 0.90	< -3	–	825	338	1,250	587	45	681
> 0.90	< -3	$> 80\%$	19	17	36	82	11	93

Table S5: High confidence ($p > 0.90$ and $z < -3$), in special with high conread coverage ($> 80\%$), and slightly more relaxed ($p > 0.50$) candidates of conserved secondary RNA structures in the PigEST data are numerated. The RNA structures were predicted in ORF-free conreads and protein-coding transcripts (conreads containing ORFs) by RNAz. The highest RNAz classified alignments of each sequence conserved EST are presented. Locally conserved secondary RNA structures are counted as loci, which are overlapping windows combined into clusters. Conreads with $p < 0.50$ have no conserved RNA structures. Predicted RNA structures in protein-coding conreads are only potential candidates of cis-acting RNA elements if they are located in an UTR. In a later step, this will be tested through their conservation to human UTRs, in which the human genome annotation serves as reference.

Conserved local RNA structures in pig, cow, human and mouse			
Organism	Number of windows		
	RNAz $p > 0.5$	RNAz $p > 0.9$	RNAz $p > 0.9$, $z < -3$
pig	29.628	12.143	1.632
cow	28.840	11.734	1.566
human	26.197	10.606	1.394
mouse	19.432	7.467	1.032

Table S6: The numbers of predicted local RNA structures (windows) in ORF-free conreads conserved in pig, cow, human as well as mouse are presented.

Reading direction of predicted RNA structures				
	RNAz criteria		conread strand	
	p-value	z-score	sense	antisense
ORF-free conread	> 0.50	–	4,964	10,565
	> 0.90	< -3	378	839
coding conread	> 0.50	–	4,206	7,687
	> 0.90	< -3	213	468

Table S7: RNA structures were predicted by RNAz on the positive and reverse complementary conread strand. The reading direction with the more evident RNA structure was predicted by RNAstrand for ORF-free conreads and coding conreads.

Human UTR homologous RNA structures - cis-acting RNA element candidates						
	RNAz criteria		RNA structure			conread
			human	UTR homolog		UTR homolog
	p-value	z-score	conserved	sense	antisense	
ORF-free	> 0.50	–	13,216	1,452	366	1,816
conread	> 0.90	< -3	1,066	146	33	177
coding	> 0.50	–	9,906	1,266	246	1,510
conread	> 0.90	< -3	580	82	18	99

Table S8: RNA structures which are located in UTRs are potential cis-regulatory RNA elements. The human conserved secondary structures in ORF-free conreads and coding conreads are mapped against the UCSC known gene annotation of human. Therefore, the more evident conread strand of a RNA structure predicted by RNAstrand is applied. The table shows the number of human UTR homologs of RNA structures (loci) on the sense as well as antisense conread strand. Obviously more cis-acting RNA elements were predicted on the positive strand, but roughly two-thirds of predicted RNA structures are located on the negative conread strand. RNA structures in protein-coding transcripts which are not aligned to an human UTR are not further analysed by the pipeline.

Known and novel miRNAs predicted by RNAmicro			
	Contigs	Singletons	loci
Known by sequence	9	5	14
Known by structure	1	2	3
RNAmicro predicted (RNAz $p > 0.9$, $z < -3$)	95	32	132
total	102	36	143
RNAmicro predicted (RNAz $p > 0.5$)	356	182	557

Table S9: The numbers of all detected microRNAs in the PigEST data are presented. These are sequence known (blast), structure known (RaveNnA) and putative novel miRNAs (classified by RNAmicro with $p > 0.9$). All RaveNnA hits are also found by sequence similarity. As input for RNAmicro are applied the alignments of all high confidence RNAz predicted RNA structures ($p > 0.9, z < -3$) in non-protein coding ESTs as well as RNAz hits with a loose threshold of acceptance ($p > 0.5$). Conserved in the cow genome are 6 of the 14 known miRNAs, of which 3 are high confidence RNAz predicted (5 with RNAz $p > 0.5$). Hence. a total of 143 loci. Note also that due to multiple hits of pig and cow, the sum of contigs and singletons does not necessarily add up with the number of loci. All high confidence RNA structures are classified as miRNA by RNAmicro plus one with slightly relaxed RNAz criteria.

ESTs which are predicted by RNAz and annotated in ncRNA databases						
	RNAz		ncRNA db			
EST name	Start	End	Start	End	Identifier	ncRNA db
rnep33c_i11.y1	1	149	23	121	MI0000695	miRBase
rplac_6675.y1	461	618	508	592	MI0001447	miRBase
reep15c_p11.y1	150	303	184	291	MI0001647	miRBase
Ss1.1-rpigcb_15937.5	40	196	73	158	MI0004756	miRBase
Ss1.1-Pig4-TMW8022F14.3	72	310	174	283	MI0002441	miRBase
Ss1.1-Pig2-138B11.5.5	450	565	476	563	MI0000865	miRBase
Ss1.1-Pig4-TMW8051D13.5	850	969	352	1091	L08437	NCBI RNAdb
Ss1.1-Ovi2-UMC-peov3-003-f02.3	236	355	745	924	AL137373	NCBI RNAdb
Ss1.1-Pig4-TMW8008G10.5	387	504	821	873	AJ012495	NCBI RNAdb
Ss1.1-Pey1-15B06.5	683	881	664	1186	TE18710	fantom3 nc
	1070	1188				

Table S10: The table presents the ESTs which are predicted by RNAz and annotated in ncRNA databases. The first 6 candidates are ncRNAs (miRNAs) and the latter 4 are cis-regulated mRNAs. The positive EST strand of all cis-acting elements is aligned to an human UTR, but RNAstrand predicts for the contigs *Ss1.1-Pig4-TMW8008G10.5* and *Ss1.1-Pey1-15B06.5* the evident RNA structure on the reverse complementary EST strand. *Ss1.1-Ovi2-UMC-peov3-003-f02.3* and *Ss1.1-Pig4-TMW8008G10.5* have the RNAz prediction and annotation in non-overlapping sequence loci. Contig *Ss1.1-Pey1-15B06.5* has two locally predicted conserved secondary RNA structures inside the annotated range.

Known and novel ncRNAs and cis-acting elements in PigEST data			
	Method	Contigs	Singletons
ncRNA	Known by sequence	20	24
	Known by structure	54	96
	Predicted	692	312
	Total	755	418
cis-acting RNA elements	Known by sequence	27	41
	Known by structure	55	47
	Predicted	243	33
	Total	315	91
Sum of all RNA structures		1,070	509

Table S11: An overview of all known and putative novel ncRNAs and cis-acting regulatory RNA elements which are detected in the PigEST data including the tRNA candidates detected by sequence and structure similarity.
